# Supplementary figures and images for: Different Species of Marine Sponges Diverge in Osteogenic Potential When Therapeutically Applied as Natural Scaffolds for Bone Regeneration in Rats
Source: J Funct Biomater. 2023 Feb 24;14(3):122. doi: 10.3390/jfb14030122 (PMC10059666; doi:10.3390/jfb14030122)

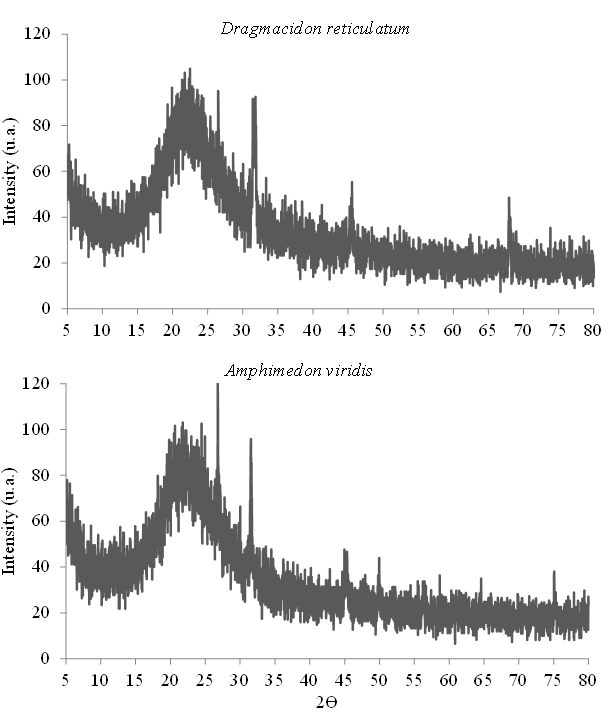

Supplement: Supplementary file 1 [file jfb-14-00122-s001.zip › figure s1.png]

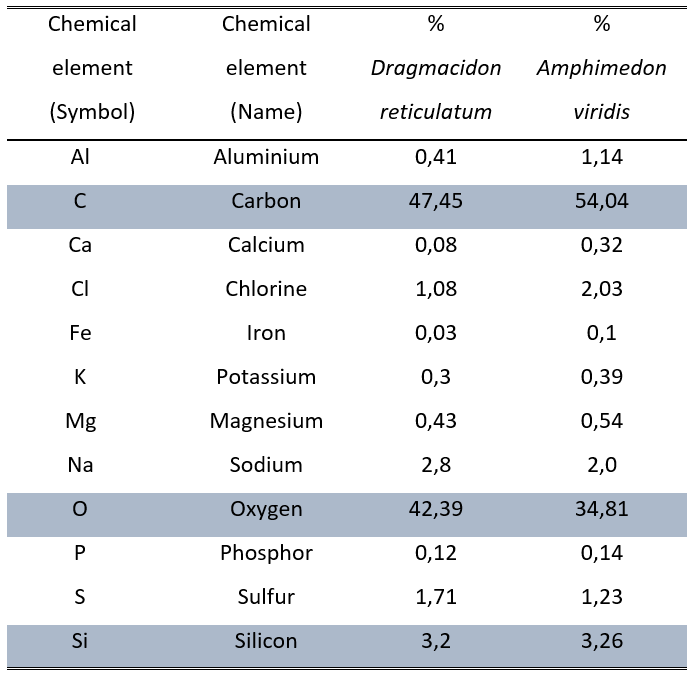

Supplement: Supplementary file 1 [file jfb-14-00122-s001.zip › table s1.png]
